# The influence of Chinese typography on information dissemination in graphic design: based on eye-tracking data

**DOI:** 10.1038/s41598-024-64964-y

**Published:** 2024-06-17

**Authors:** Weilong Chen, Jiqiang Yang, Yiluo Wang

**Affiliations:** 1 College of Design and Innovation, Zhejiang Normal University, Jinhua, 321000 China; 2 College of Design and Innovation, College of Education, Zhejiang Normal University, Jinhua, 321000 China

**Keywords:** Graphic design, Chinese characters, Layout, Information dissemination, Human behaviour, Short-term memory

## Abstract

The arrangement of Chinese characters has a significant impact on the visual effect and information dissemination in graphic design. In traditional Chinese layout, vertical arrangement of characters is predominant, but in recent times, there has been a gradual transition towards horizontal arrangement. To compare the influence of different character arrangement forms on visual meaning generation and information dissemination, This study employed an eye-tracking experiment to investigate two common Chinese character layouts in posters—horizontal and vertical, and collected data such as eye-tracking heatmap, pupil diameter and eye-tracking trajectory map. Based on objective eye-tracking data, combined with post-test interviews and questionnaire surveys, it was found that vertical character arrangement in Chinese typography is more effective in attracting visual attention and facilitating the expression and stimulating interest in viewing/reading under the premise of meeting formal requirements, which may provide guidance and inspiration for the practical application of Chinese characters in layout design, advertising design, packaging design, exhibition design, UI design, and other related fields.

## Introduction

Text plays an important role in the fully developed society of commodity economy by disseminating information about products, culture, even knowledge, and text arrangement is part of the layout design, guiding the line of sight when reading, which has a significant impact on the reading experience^1^. For example, posters have significant functions in political propaganda, spiritual guidance, and information dissemination. Graphic design encompasses various aspects such as font design, logo design, illustration design, packaging design, advertising design, display design, and layout design. Its essence lies in using visual symbols and forms to aesthetically express the connotation and information to be conveyed in society. The fundamental purpose of design art is to convey information, and text is an important medium for conveying information. Visual communication design, which is primarily based on typography, is a public art form. The process largely involves the manipulation of text and graphics^2^. As an ancient hieroglyphic script, Chinese characters have a unique stroke structure and require special design considerations in layout.

Visual elements such as graphics and text form a symbolic system, which imparts specific meanings to them in overall layout. As creative symbols, they have a clear reference function in graphic design works, playing a necessary role in information dissemination^3^. In this process, text has evolved into a graphic symbol with additional information—rather than just lexical and linguistic symbols, and it has its own value in composition and layout. As the basic component, characters represent points, words and sentences represent lines, and text, as a visually understandable element, appears in forms of dots, lines, and surfaces. It becomes part of the layout and even the integration, achieving a unique composition style that combines text and images^4^.

## Literature review

### The influence of layout design on the conveyance of graphic meaning in Chinese characters

The layout design of Chinese characters has different characteristics and rules compared to English, as Chinese characters have unique font structures and character-making methods. In contrast, English words are formed by combining the different combinations of 26 letters, while Chinese characters are created based on strokes resembling lines. Some researches^5^ pointed that Chinese characters have more structural details and are visually more complex in form than alphanumeric ones. Earlier studies compared the effects of vertical and horizontal layouts of Chinese and English texts on reading performance, such as the research by Chen & Carr^6^, they examined the reading and editing abilities of Chinese students when presented with Chinese characters arranged vertically and English words arranged horizontally. The results showed that when Chinese characters are arranged vertically, all four groups of students were able to read and edit faster and more accurately. In contrast, when English letters and Arabic numerals were presented horizontally, better results were typically achieved. Some scholars have also compared the influence of character spacing in Chinese and English road signage on driver sight distance (reading), as well as the similarities and differences in eye movements when reading printed texts in their native language systems. Sun^7^ found through recording the eye movements of participants that modern readers are more accustomed to reading horizontal text, which is contrary to Chen & Carr’s result. The possible reason is that most publications in modern China have gradually abandoned the traditional vertical layout and adopted a more popular horizontal layout. However, in terms of practical signage design, vertical layout of Chinese characters is more advantageous for user perception. Song^8^ have found through experimental research that traditional Chinese characters displayed on outdoor signage in mainland China which were arranged vertically increase potential consumers’ perceptions on consumer scenarios. Huang et al.^9^ conducted empirical research on the impact of vertical and horizontal arrangements of Chinese characters on reading performance, including reading duration, memory effectiveness, and cognitive load. The findings suggest that readers tend to read faster when text is presented in a horizontal layout, and that horizontal reading is more effective for remembering long texts compared to vertical reading. However, for short texts, the influence of text orientation on memory is not significant. These findings provide valuable insights into the impact of text orientation on Chinese typography and offer suggestions for better layout design, ultimately help readers improve their reading efficiency and information acquisition.

As being discussed above, Most studies on text layout focus on the impact of the arrangement of characters in horizontal and vertical directions on readability, legibility or visual perception, specifically in the following aspects:

1. Typically focuses on the influence of text orientation on reading speed^10^, with subjects including university students, drivers, and individuals of different ages^11,12^. The main research material is text, with variables such as text length, spacing, and orientation being examined^13^. Some studies also examine the impact of font size and style (traditional Chinese characters) on readability^14–18^, with Kai or Hei styles being found to be most suitable for reading on mobile screens^19^.
2. Primarily conducted using smartphones^5^ and printed media.
3. Empirical studies are predominantly conducted, some results indicating that vertically arranged Chinese characters have better communication effectiveness than horizontally arranged ones—especially on short text. However, some studies suggest that horizontally arranged characters may be faster than vertically arranged ones in terms of reading speed but there is no significant difference in memory effects between the two arrangements.
4. Some studies compared the impact of different arrangements of Chinese characters on information dissemination effectiveness, for example, the use effect in practical life and commercial products such as restaurant signs, traffic signs, etc.

However, most of the experimental materials used in these studies are primarily text. In modern society, the means of information dissemination are no longer limited to traditional text, but rather more focused on the integration of images and text. We live in an environment where both image and text coexist. Although text often takes a subordinate role, this does not diminish the importance of text in information communication. In the era of visual communication, where images are the primary means of expression, the layout of text significantly impacts reading and information conveying, expressive typography can add emotional emphasis to a design’s message but may interfere with legibility^20^. Therefore, when designing imagery Chinese characters and graphics together in typography, what differences exist between vertical and horizontal arrangements in terms of their impact on information dissemination? How to combine image and character effectively for efficient information transmission? It is an important issue that needs to be considered in the visual era.

### Arrangement of Chinese characters: perspective of combining character and image

In China, the traditional layout has always been vertical arrangement of characters^21^. The issue of horizontal and vertical arrangement of Chinese characters can be traced back to the development of calligraphy and printing technology in ancient China. In traditional Chinese calligraphy, characters are mainly written vertically, with horizontal writing being less common.With the introduction of Western classics in modern times, there arose a contradiction in layout between horizontal arrangement of foreign languages, Arabic numerals, equations, formulas, Western symbols and the vertical arrangement of Chinese characters. As a result, the Chinese intellectual community began advocating for horizontal arrangement of Chinese characters^22^. In the development of printing technology, however, horizontal arrangement of characters became mainstream for the convenience of typesetting and reading. In the application of modern computer technology, people are more accustomed to using horizontal arrangement to display Chinese characters, as modern computers primarily use Western languages, which are horizontally arranged. However, in certain special cases, such as traditional art design, calligraphy exhibitions, Chinese typesetting, cultural books, etc., Chinese characters need to be vertically arranged to showcase the characteristics and aesthetics of traditional culture.

The purpose of graphic design is to convey information and plan the position, proportion, and relationship of visual objects in a two-dimensional space. In the aesthetic culture and information dissemination based on graphic design, Chinese characters play an important role in both an important form and key means of information transmission. This is because Chinese characters have both functional and aesthetic attributes since inception, possessing practical value and aesthetic value. In almost all forms of graphic design, character is an important and indispensable component because it can convey clear and specific information. Only through corresponding text people can grasp the core content, while images need to imply and echo the theme. When people first see a picture, they experience strong and direct visual sensations, which are achieved through images, tones, and composition. However, the information that needs to be conveyed to readers must be realized through characters^23^. In graphic design, text, graphics, and colors are three major forms of expression and effective carriers of information. The combination of text and graphics has a significant impact on the visual representation and even the generation of visual meaning^24^. From the perspective of the relationship between characters and graphics, there are roughly three types of relationships:

1. From the perspective of content presentation, the image takes the lead while the text generally plays a subordinate role, but serves as the finishing touch. The image is the main visual element that attracts viewers’ attention, while the text often highlights the theme, purpose, and meaning of the work to effectively convey its information and essence^25^. In terms of layout, it is often arranged horizontally or vertically. The text is not only a visual element but also a tool for expressing content.
2. From a visual perspective, text and image complement each other, mutually enhancing and blurring the line between them, forming what Klee Bell calls “formal significance”^26^. For example, some poster works directly derive from the transformation of letter forms, or the graphicization of characters. In such cases, graphic works often start from the design of typography, letter forms, and sizes, aiming to achieve a perfect unity and coordination between text and graphics in terms of their forms, pursuing aesthetic beauty. For instance, the aesthetic concept of “the same origin of calligraphy and painting” in China explores the visual elements of text and graphics, establishes connections and transformations between them based on visual grammar and structures, and refines, combines, and extends their forms, meanings, and artistic charm.
3. From a functional perspective, characters and images have their own roles and clear divisions. The functional differentiation between characters and graphics in two-dimensional works is generally manifested by clearly delineating them into separate areas (panels) on the composition to fulfill their respective functions. Sullivan advocated for “form follows function”, while Frank Lloyd Wright goes even further, believing that “function and form are one and the same, and it is fundamentally impossible to completely separate them”. Béla Balázs also holds that “function comes first, decoration comes second”, and his designs highly align with the harmony between functionality and decoration^27^. Similarly, in the works of Bradley, a representative of the American ‘Art Nouveau”(New Art) movement, illustrations and text are enclosed in simple rectangular frames, thus having distinct functional areas: illustrations and text are separate without any confusion^28^. These are the so-called functionalist viewpoints in history, which extensively embrace minimalist designs. In contemporary graphic design works, we can still find inheritance and development of this style and characteristics.

Of course, We need not to view character and image from a dualistic perspective. On the contrary, from aesthetic and communication perspective, they should be integrated, serving the visual communication needs of the graphic work in terms of form, content, and even functionality. However, practical questions such as what the graphic design work is promoting, what to design, and how to design it are issues that designers need to consider during the creative process. Text plays a significant role as an important means of expression and medium for designers. Since ancient times, pictographic writing systems have been created in human civilization, and although the form of Chinese characters has evolved, their effectiveness in conveying information remains unchanged. In graphic design, visual elements such as graphics, text, and colors form a symbolic system, and Chinese characters, as the earliest symbols used for communicating information and meaning, have become tools. The arrangement must consider the relationship between Chinese characters, graphics, and colors as a whole. At the same time, Chinese characters, as graphic elements themselves, are also utilized by many designers. The Chinese characters themselves possess aesthetic beauty in their form, and the use of pictographic characters has been evident in oracle bone script, adhering to the principles of visual aesthetics in their structure to this day^29^.

The arrangement of characters is the appearance of text as points, lines, and surfaces in layout, becoming a part of or even the entirety of the layout. As a square-shaped font, Chinese characters are primarily composed of horizontal and vertical strokes. Horizontal strokes appear most frequently in Chinese characters and play a crucial role in their structure, creating tension between the height of the upper and lower parts of the square. Generally, horizontal strokes are treated thinner, and they are usually not filled to the top of the square to adjust the visual center of gravity. On the other hand, vertical strokes are thicker and provide essential “structural support” to the entire font. In contrast to horizontal strokes, vertical strokes more often fill the outline, causing Chinese characters to be slightly longer within the square and creating a trend of vertical development. In Japanese typography, which preserves a large number of Chinese characters, vertical text layouts are far more prevalent than horizontal ones, forming a distinct contrast with the predominantly horizontal layout of English^30^. As a phonetic script, English is visually more suitable for various forms of layout, such as serif or sans-serif fonts. Experienced designers know that the same line of text will have different visual effects when arranged horizontally or vertically in English or Chinese characters. Therefore, in graphic design, the arrangement of Chinese characters is an important aspect of design practice.

Text and image are the two major components of any visual medium. The quality of textual arrangement directly influences the visual communication effect of a layout^31^. Therefore, in graphic design works, the vertical and horizontal arrangement of Chinese characters have a certain impact on visual communication. In design practice, designers often arrange Chinese characters based on their experience and from the perspective of visual aesthetics. In some cases, when arranging Chinese characters, they may face ambiguity or even a dilemma. For example, from a formal standpoint, vertical arrangement is more aesthetically appealing, but from a functional perspective, horizontal arrangement can also achieve the purpose and function of information dissemination. Faced with commercial design demands, designers need to provide objective justifications to convince clients to adopt corresponding design solutions. In some cases, it is necessary to make judgments based on aesthetic values from a subjective standpoint, but this alone is not sufficient. Therefore, by utilizing data obtained from eye-tracking experiments and questionnaires, and through organizing and analyzing this data, the issue of vertical and horizontal arrangement of Chinese characters can be interpreted from a more objective and scientific perspective.

Based on the above analysis and discussions, this paper formulates the following two hypotheses:

1. When conveying information through both images and text which is single-line, horizontally arranged Chinese characters which are arranged parallel to images horizontally result in higher readability compared to vertically arranged Chinese characters.
2. In the context of conveying information through both text and images, a vertical text layout stimulates greater reading/viewing interest in viewers compared to a horizontal text layout.

## Method

This study was approved by the ethics committee of Zhejiang Normal University, and the experiments were performed in accordance with the approved guidelines.

### Materials

**Figure 1:**
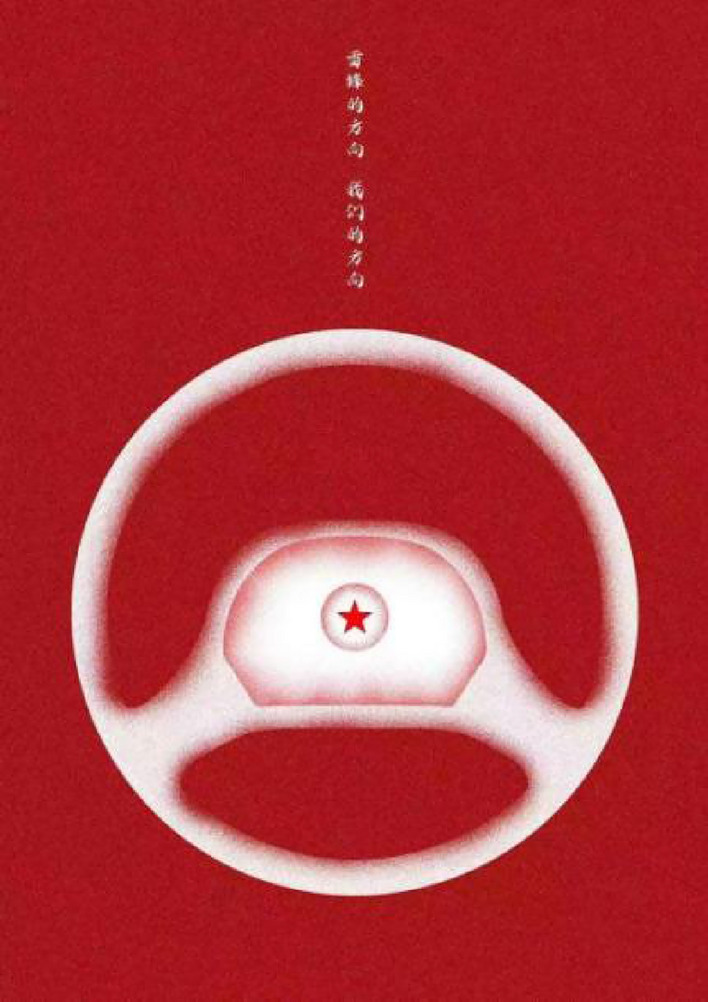
Vertical group poster.

**Figure 2:**
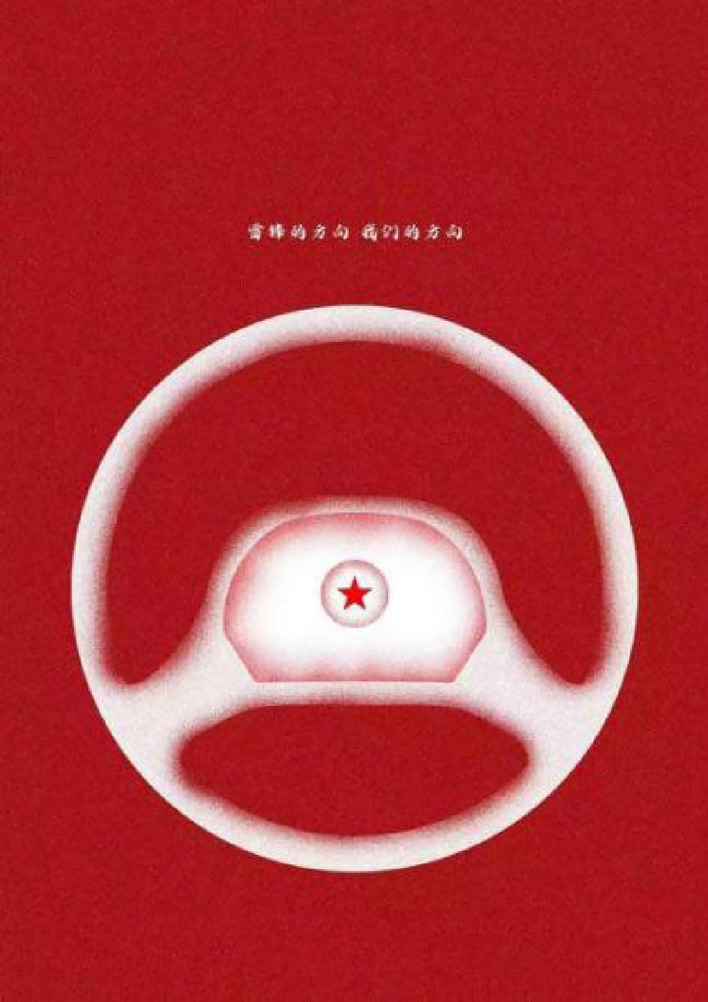
Horizontal group poster.

We used two posters (see Figs. 1 and 2) as the experimental material to conduct an eye-tracking experiment. Both of the two slides are consisted of ten Chinese characters and an image below the text, and they have the same resolution(size) of 1280*1810 pixels. The only difference is the layout of the Chinese characters—vertical layout (Fig. 1) and horizontal layout (Fig. 2).

Words on the posters are “雷锋的方向 我们的方向” which means “Leifeng’s direction is our direction”. The two groups of Chinese characters were identical in font size-which is 22pt, spacing-which is semi-character spacing and the typeface is “iSide-iFonts” which is similar to “semi-cursive script”and more fluid than standard script;the only difference is layout. The setting of interword spacing referenced the study by Hsu and Huang^32,33^ on adult reading of Chinese characters as well as the study by Grobelny and Michalski^34^.The horizontal arrangement of text refers to the arrangement of text and images on parallel horizontal lines, rather than on the same line. The selection and use of fonts in posters design is different from that of typical printed texts, such as books and newspapers, so we did not choose the Hei or Kai font in our materials.

### Apparatus

The eye-tracking instrument used in the experiment was the Tobii Pro Fusion eye-tracking system, sample rate 250 Hz, the distance between the eye tracker and the participant’s eyes is maintained at 60–65 cm (23–26 inches), with the line of sight centered on the middle of the screen(this research is based on screen project).

### Subjects

The experimental subjects were consisted of 40 individuals randomly selected from students in a Chinese university, each with normal vision suitable for eye-tracking experiments and they can correctly read Chinese characters in experimental materials, with 20 individuals in each group (9 males and 11 females). The age range was 20 to 24 years old(*M* = 22.1, *SD* = 1.4).

Before the experiment began, the experimenters fully informed the participants of the experimental process, the collected data, and the purpose of the data, and obtained the consent of each participant. All participants signed an informed consent form prior to the experiment.

### Experimental design

In this study, we employed a 2 × 2 between-subjects experimental design where the independent variable included two levels of Chinese character layout: vertical and horizontal. Gender was considered as a potential influencing factor. Participants were randomly assigned to one of two groups: the Vertical Group, who viewed posters with text arranged in a vertical orientation (see Fig. 1), and the Horizontal Group, who viewed posters with text arranged horizontally (see Fig. 2). The primary aim of the study was to investigate whether the different character layouts affect fixation duration and to examine if this effect varies between males and females. Additionally, other eye-tracking data such as pupil diameter and eye movement trajectory were collected to obtain more comprehensive visual processing information.Both of the two groups have never seen the experimental materials before.

### Procedures

The experimental procedure was as follows: the experimenter informed the subjects of the experimental process and requirements. The subjects watched the experimental material on the eye-tracking device for 5 s referred to Ma & Chuang’s research^35^, and the experimenter saved the experimental data for each subject. Through the eye-tracking experiment, we obtained data such as eye-tracking heatmaps, pupil diameter, and eye-tracking trajectory map of both groups of subjects.After the experiment, a questionnaire survey was conducted on both groups of subjects. The questionnaire consisted of two questions: a multiple-choice question: “Please select the content you saw on the screen” and a fill-in-the-blank question: “Can you recall the text content displayed on the screen and write it on the line?”.

## Experimental results and discussion

### Heatmap and results of the analysis of variance (ANOVA) of text and image

**Figure 3:**
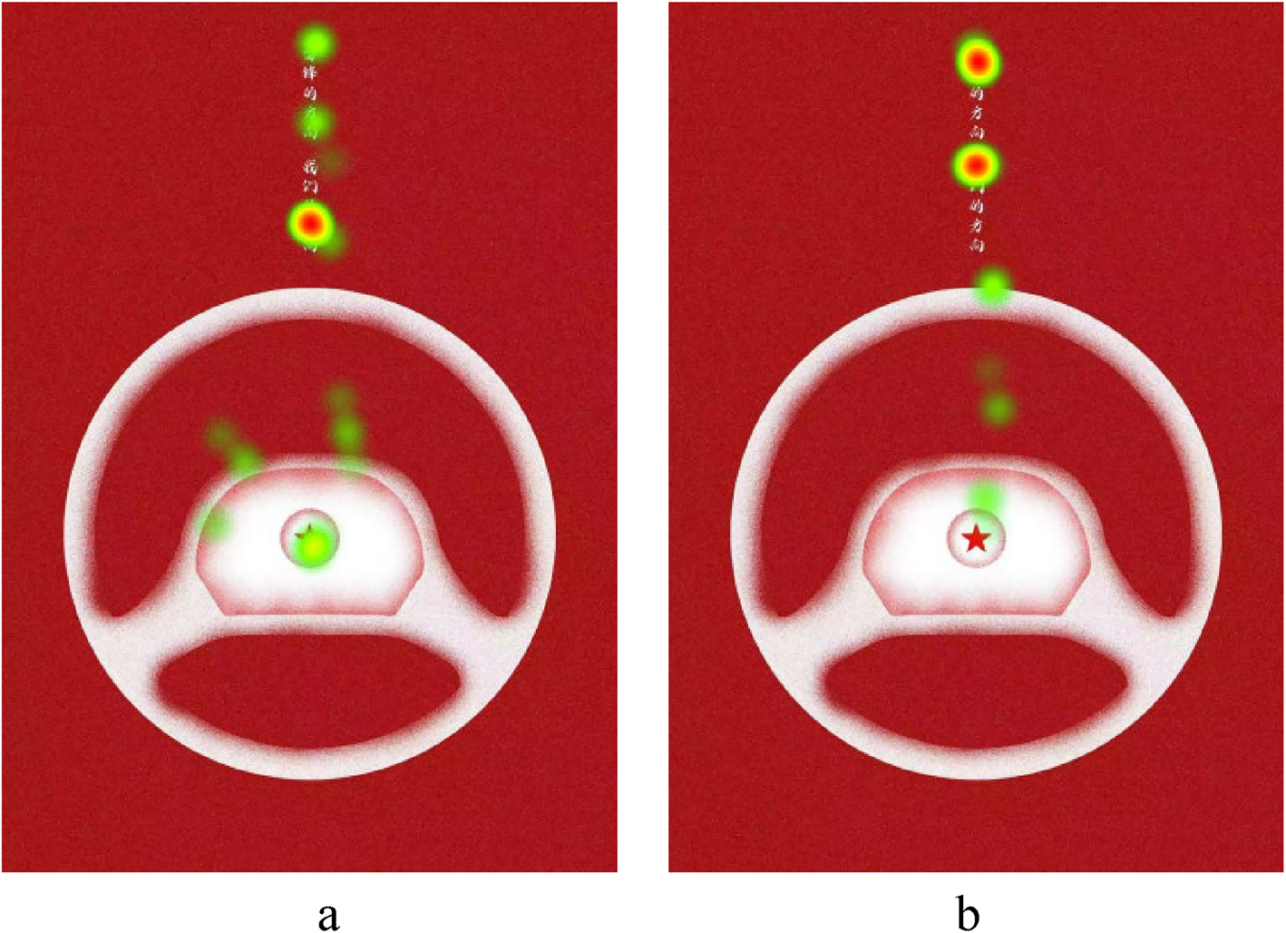
(**a**) Heatmap of subject, (**b**) heatmap of subject.

**Figure 4:**
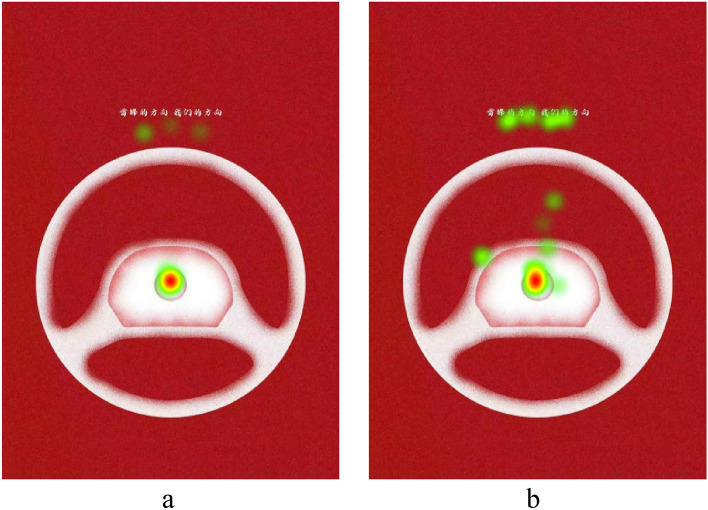
(**a**) Heatmap of subject, (**b**) heatmap of subject.

Eye-tracking heatmaps are visual representations of the average gaze time of participants on specific locations in an image. The darker the color, the longer the gaze time. Due to space limitations, we present the heatmaps for a subset of subjects from both the experimental and horizontal groups. see Fig. 3a,b of vertical group, and Fig. 4a,b of horizontal group, From the heatmaps, it is evident that participants in the vertical group spent significantly more time looking at the text portion compared to the horizontal group (some participants from the horizontal group showed minimal or no gaze time on the text portion, as seen in Fig. 4a). This indicates that the text layout in the vertical group’s poster attracted more attention.

**Figure 5:**
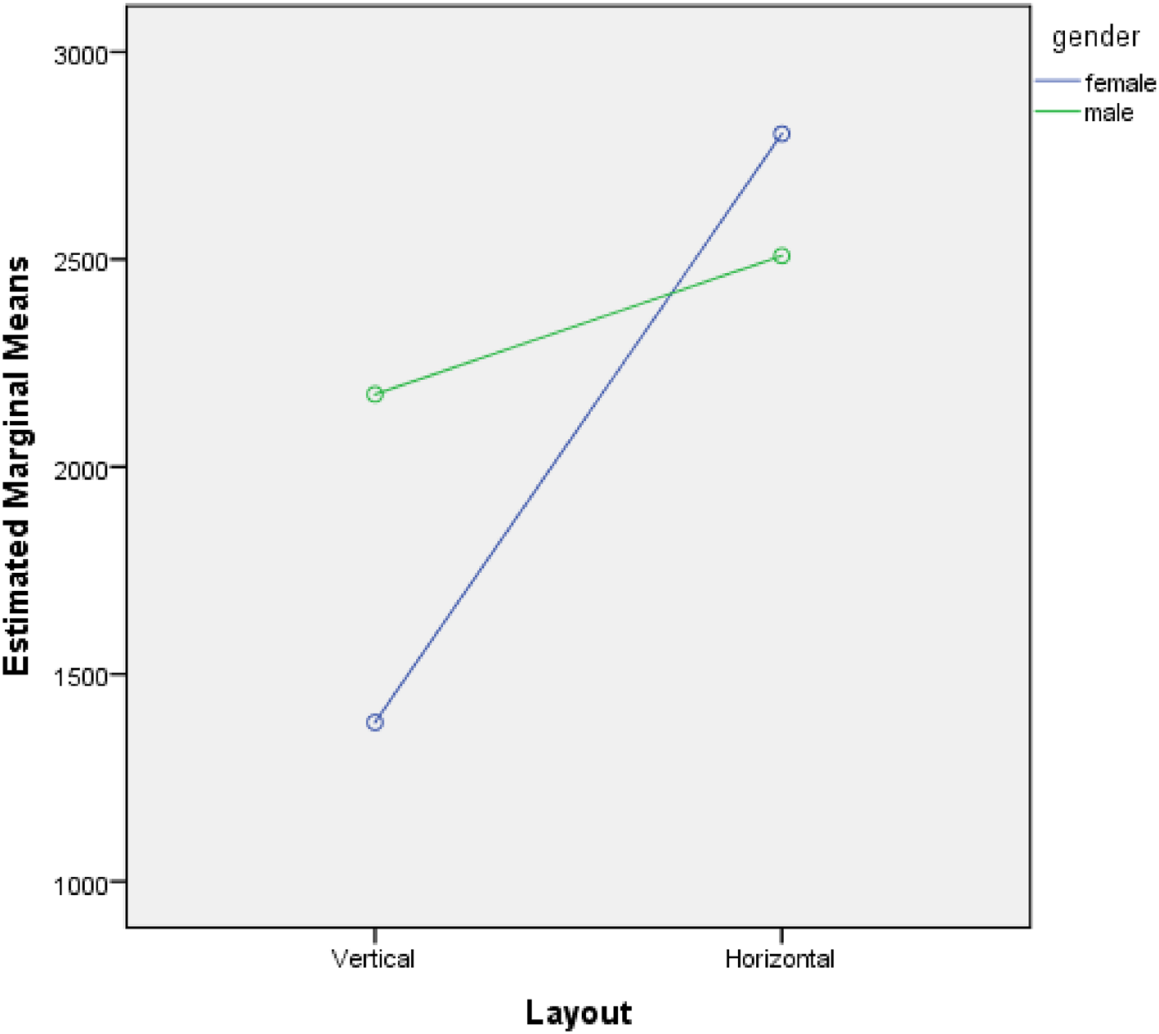
Estimated marginal means of image.

The results of the analysis of variance (ANOVA) indicated a significant main effect of text layout, *F*_(1,36)_ = 11.926, *p* = 0.001, with participants’ fixation duration significantly higher when the text was arranged vertically(*M* = 2655.50) compared to horizontally (*M* = 1799.10). The main effect of gender was not significant, *F*_(1,36)_ = 0.958, *p* = 0.334. However, there was a significant interaction effect between text layout and gender, *F*_(1,36)_ = 4.571, *p* = 0.039. Simple effects test revealed that when the text was arranged vertically, males *(M* = 2174.60) had a significantly longer fixation duration on image of the poster than females (*M* = 1383.60), *t*_(14.868)_ = -−3.274, *p* = 0.005. When the text was arranged horizontally, there was no significant difference in fixation duration between males and females,*t*_(18)_ = 0.659, *p* = 0.518. See Table 1 and Fig. 5.

**Table 1:** Results of the Analysis of Variance (ANOVA) of image fixation duration.

Sources of error	df	Mean square	F	Sig	Partial Eta squared
Gender	1	617,025.600	0.958	0.334	0.026
Layout	1	7,680,769.600	11.926	0.001	0.249
Gender * Layout	1	2,944,147.600	4.571	0.039	0.113
Error	36	644,055.989

**Figure 6:**
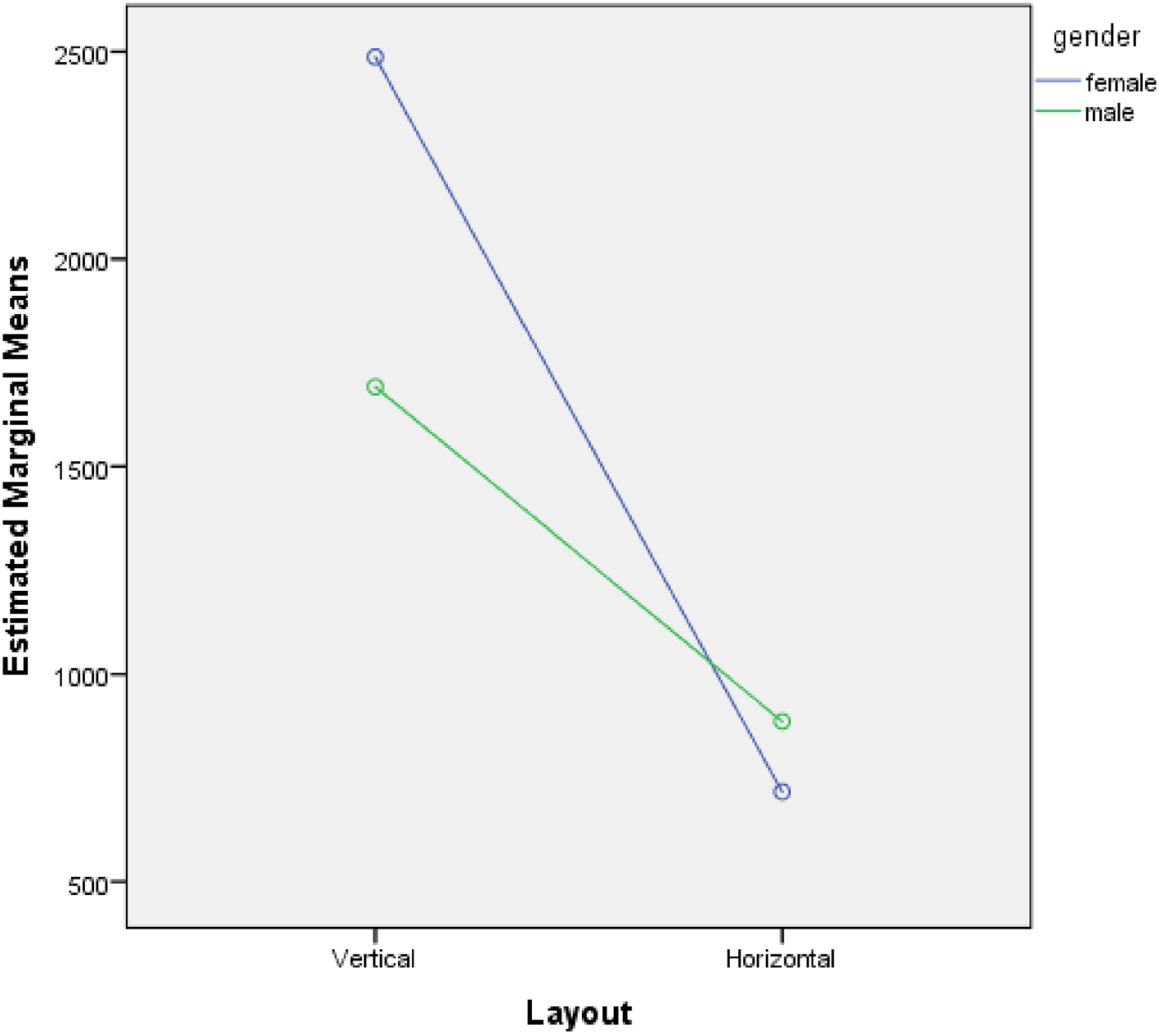
Estimated marginal means of text.

The results of the analysis of variance (ANOVA) indicated a significant main effect of text layout, *F*_(1,36)_ = 61.184, *p* < 0.001, with participants’ fixation duration significantly higher when the text was arranged vertically(*M* = 2089.55) compared to horizontally (*M* = 801.50). The main effect of gender was not significant, *F*_(1,36)_ = 3.605, *p* = 0.066. However, there was a significant interaction effect between text layout and gender, *F*_(1,36)_ = 8.584, *p* = 0.006. Simple effects test revealed that when the text was arranged vertically, females (*M* = 2487.10) had a significantly longer fixation duration on text than males (*M* = 1692.00), *t*_(18)_ = 2.607, *p* = 0.018. When the text was arranged horizontally, there was no significant difference in fixation duration between males and females,*t*_(18)_ = -1.365, *p* = 0.189. See Table 2 and Fig. 6.

**Table 2:** Results of the analysis of variance (ANOVA) of text fixation duration.

Sources of error	df	Mean square	F	Sig	Partial Eta squared
Gender	1	977,500.225	3.605	0.066	0.091
Layout	1	16,590,728.025	61.184	0.000	0.630
Gender * Layout	1	2,327,580.025	8.584	0.006	0.193
Error	36	271,159.881

The horizontal arrangement of Chinese characters tends to highlight the horizontal strokes of the characters, creating a “horizon” effect that brings a sense of stability. However, compared to static and stable objects, dynamic or objects with a tendency to move are more likely to attract human attention^36^.

Human eyes exhibit the phenomenon of the horizontal–vertical illusion (HVI), where the vertical lines of a shape, such as the ∟ symbol, appear longer than the physically equivalent horizontal lines. Schiffman and Thompson^37^ pointed out in their research on the horizontal–vertical illusion that the eye movement hypothesis suggests that greater effort is required for vertical movements compared to horizontal movements, and this effort translates to a perception of greater visual length to some extent. According to this hypothesis, the same amount of vertically arranged Chinese characters may require more attention time compared to horizontally arranged ones, as vertical distances require more effort than equivalent horizontal distances.

### Pupil diameter result

#### Average pupil diameter data

Through eye-tracking experiments, pupil diameter data of participants during the experiment can be obtained, as shown in Table 3. Hess & Polt’ research has found that an increase in pupil size accompanies the observation of emotionally soothing or interesting visual stimuli^38^.

**Table 3:** Mean and standard deviation of average pupil diameter(Unit: millimeters).

Pupil diameter	Layout	subjects	Mean value (M)	Standard deviation (sd)
Average_pupil_diameter.Image	Vertical	20	4.085	0.393
	Horizontal	20	3.014	0.320
Average_pupil_diameter.Word	Vertical	20	3.672	0.461
	Horizontal	20	2.954	0.347

Table 4 presents the independent samples t-test for the average pupil diameter of two groups when viewing text and graphics. From Table 4, we can see that *p* = 0.024 < 0.05, indicating a significant difference in the average pupil diameter of the experimental and horizontal groups when viewing text in the poster. The Wilcoxon rank-sum test yielded *p*-values of 0.009 and 0.047 (see Table 5), both smaller than 0.05, also suggesting a significant difference in the average pupil diameters between the two groups when viewing text and graphics. Furthermore, from Table 4, we can also observe an independent samples t-test for the average pupil diameter of the two groups when viewing graphics, with *p* = 0.001 < 0.05, indicating a significant difference. A possible explanation for these findings is that the vertical arrangement of Chinese characters expands the visual space of the poster in the vertical direction, thereby increasing the amount of information and the meaningfulness of the poster. This, in turn, leads to a greater physiological response from the viewers’ eyes. Additionally, it also generates interest and motivation in the audience regarding the conveyed information of the poster.

**Table 4:** Independent samples t-test.

Pupil diameter	t	Degrees of freedom	Significance level(two-tailed)		95% confidence interval	
				CI Lower		CI Upper
Average_pupil_diameter.Image	4.731	8	0.001	0.549		1.594
Average_pupil_diameter.Word	2.780	8	0.024	0.122		1.314

**Table 5:** Wilcoxon rank-sum test for pupil diameter (non-parametric analysis).

Pupil diameter	Mann–Whitney U	Wilcoxon W	Z	Significance level(two-tailed)
Average_pupil_diameter.Image	0.000	15.000	− 2.611	0.009
Average_pupil_diameter.Word	3.000	18.000	− 1.984	0.047

By comparing the physiological data of average pupil diameter, it can be observed that the participants in the vertical group were more engaged in viewing graphics compared to those in the horizontal group. The vertical arrangement of textual elements in the poster also played a facilitating role in directing attention towards the graphics. This emphasizes the importance of considering both text and graphics as a cohesive entity and taking a systematic approach when designing visual materials.

Comparing the physiological data of average pupil diameter, it can be observed that the subjects in the vertical group are more engaged than those in the horizontal group when viewing the graphics. Therefore, the vertically arranged text on the posters also facilitates attention towards the graphics, indicating the necessity of considering text and graphics as a whole and in a systematic manner in graphic design.

#### The number of fixations on image and text

The number of fixations on image and text while viewing the poster was counted for the participants, as shown in Table 6. The differences in the number of fixations on text and image during the process of viewing the poster were examined using the Wilcoxon rank-sum test, and the results of the analysis are presented in Table 7.

**Table 6:** Mean value of the number of fixations.

Groups	Fixation area	Rank of mean value	Sum of ranks
Vertical-text	Image	7.20	36.00
	Text	3.80	19.00
Horizontal-text	Image	7.50	37.50
	Text	3.50	17.50

**Table 7:** The Wilcoxon rank-sum test for the number of fixations.

Groups	Mann–Whitney U	Wilcoxon W	Z	Significance level(two-tailed)
Vertical-text	4.000	19.000	− 1.844	0.065
Horizontal-text	2.500	17.500	− 2.102	0.036

In the vertical-text group, there was no significant difference between the number of fixations on text and image (*p* = 0.065). However, in the horizontal-text group, the number of fixations on image was significantly greater than the number of fixations on text (*p* = 0.036), this indicates that under the horizontal conditions the subjects focused more on image, and the text didn’t arouse their attention, this can be seen from the items they recalled significantly lower than the vertical group after experiment.

### The eye movement trajectory map

**Figure 7:**
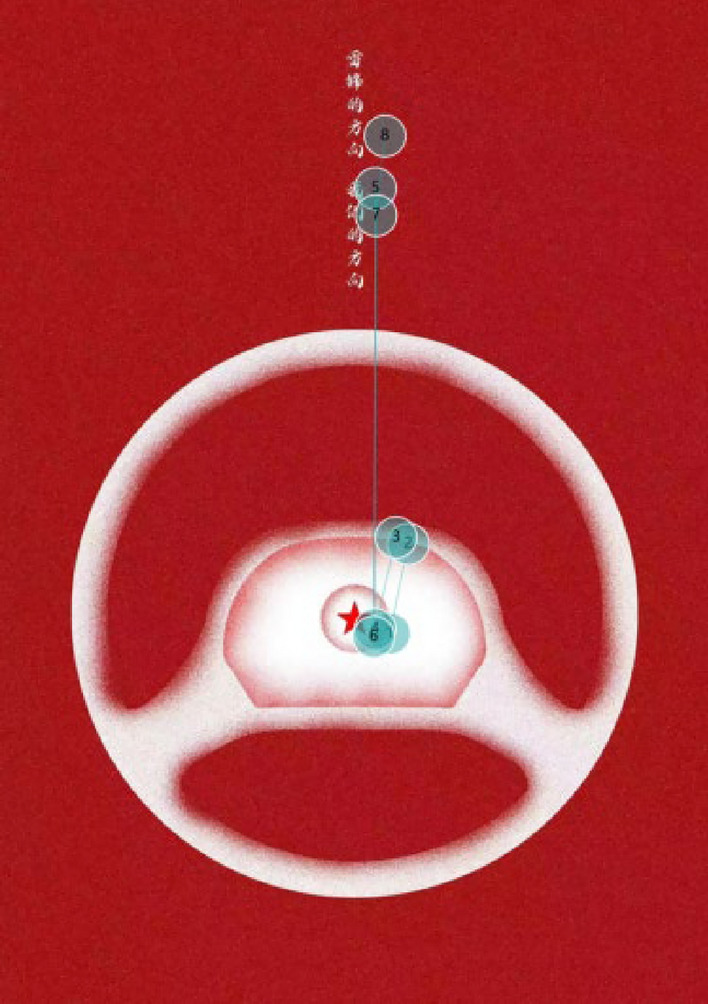
General trajectory of vertical text.

The eye movement trajectory map records the movement trajectory of the eyeball on the viewed object and the movement between the graphics and Chinese characters during the viewing process. By analyzing the eye movement trajectory, we may discover:

1. Both the vertical group and horizontal group, the starting point of the subjects’ eye movement trajectory is both the graphics in the poster, and the majority of eye movement is concentrated around the position of the “pentagram” in the poster. This suggests that for users, graphics are often noticed before text. After viewing the graphics, the audience seeks to understand the meaning conveyed by the graphics, that is, they look for other information (such as text) on the screen to interpret the visual content.
2. Subjects in the vertical group exhibits more saccades which re-view between the graphics and text, meaning they repeatedly shifted their gaze between the graphics and characters. In other words, after viewing the graphics, they would then focus on the text, and then return from the text area to the graphics area (see Fig. 7). In Fig. 7, the trajectory from position ④ to position ⑤ reflects the gaze shifting from the graphics area to the text area, while the trajectory from position ⑤ to position ⑥ indicates the return from the text area to the graphics area. The trajectory from position ⑥ to position ⑦ and then to position ⑧ reflects another return from the graphics area to the text area. According to Berlyne’s research^39^, He found that while viewing a picture, initial eye saccades were long and fixations were short, typically within the first few seconds. Furthermore, vertically arranged Chinese characters are more pictorial and embody the inherent homology of Chinese characters in calligraphy and painting. As a result, subjects in the vertical group exhibited more saccades than those in the horizontal group.

**Figure 8:**
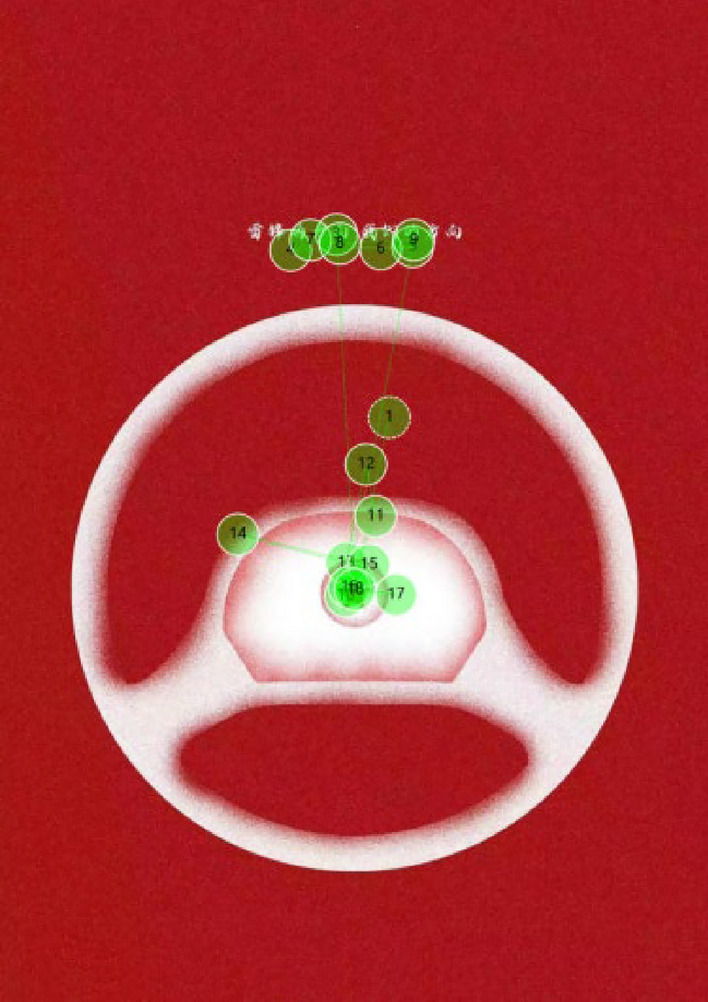
General trajectory of horizontal text.

While there was almost no such “re-viewing” phenomenon between graphics and text areas in horizontal group subjects, there was “re-viewing” just within the text area or graphic area(see Fig. 8). In Fig. 8, the subjects viewed the text area in a normal sequential order from position ④ to position ⑤, but the process from position ⑦ to position ⑨ involved re-viewing. The vertical group did not exhibit this phenomenon. This suggests that during the 5-s observation period, the horizontal group spent less time fixating on the text, which may explain the different recall performance of the two groups in the post-test survey.

After each participant finished observing the poster, a questionnaire was immediately administered.This study assumed that each item had the same weight, so a simple encoding method was included to test the recall of five items(Five-pointed star, steering wheel, military cap, and two sets of Chinese characters), with 1 point for each item. The first question consisted of three items, and one point was awarded if the item was answered correctly. For the second question, participants were instructed to write down the text they have seen on the posters. If they wrote either “雷锋” or “方向” which were thought to be the keywords on the poster, score 1 point, both score 2 points, neither score 0. The total score for vertical group was 87, and for horizontal group it was 70. An independent samples t-test was conducted, and the results showed that there is a significant difference in the mean between the two groups (*t*_(38)_ = 3.569, *p* = 0.001). Therefore, under the condition of this study,different layouts of Chinese characters on posters have a considerable impact on the effectiveness of information dissemination.

## Conclusions

The font size, font type, color, and other factors of Chinese characters in graphic design can have impacts on information dissemination. However, this experiment strictly controlled the influence of these variables and only focused on the impact of horizontal and vertical arrangement on information dissemination of posters. As can be seen through the analysis of eye-tracking experimental data, both horizontal and vertical Chinese characters have varying degrees of impact on the understanding of posters, with some significant differences observed.

1. Vertical Chinese characters are more likely to attract viewers’ attention. Experimental data shows that the combination of vertical Chinese characters and image forms a visual “center of interest”, making it easier for text and image to generate visual space and promote the generation of meaning and the transmission of information. Therefore, in media communication design such as web page design and mobile app layout design^40,41^, it is advisable to consider using vertical arrangement as the main format in order to accurately convey meaning.
2. Under the condition of single line of text and images,vertical arrangement of Chinese characters establish a connection between graphics and text, making it easier to stimulate viewers’ thinking and facilitate the information dissemination in visual works as is shown in post-test. In layouts that combine both text and graphics, the eye movements of subjects, such as saccades and fixation time, differ from those in high-density information content layouts as Nayak & Karmakar^42^ noted that long saccade and short fixation eye movements were observed in high-density information contents. In graphic design, text usually constitutes a small amount of low-density content focused on conveying essential information. Text and graphics work together to convey visual meaning, with text often acting as a guide that highlights the main points. This is because text tends to communicate meaning more directly and clearly than graphics.
3. Text appears in forms of dots, lines, and surfaces as a visually understandable element. In this study, the arrangement of single-line text (as lines) and images yielded different reading effects and influenced visual perception and comprehension. Compared with horizontally arranged ones, vertically arranged Chinese characters help to expand the visual space of the composition vertically. Except for single characters, most Chinese characters are compound characters composed of multiple components. Although compound characters are complex in form, more than half of them are pictophonetic characters with left–right or top–bottom structures^43^. In the structure of Chinese characters, vertical strokes play the role of “standing tall”, while horizontal strokes play the role of beams. Vertical arrangement of Chinese characters can bring about a more “dynamic” effect and trend of movement^44^, creating a more pronounced “line” effect which is advantageous for expanding limited two-dimensional visual space. With the development of information technology, the application of Chinese characters in new media design needs to be arranged from the perspective of information presentation, reflecting the dynamic changes of text and graphics—thereby forming a new paradigm in the field of visual information dissemination design^45^.

In design practice, the layout method used for horizontal and vertical arrangement of Chinese characters will have an impact on the communication of information. Vertical arrangement of Chinese characters is relatively narrow in width and is suitable for concise and brief information dissemination, such as vertical advertisements and business card designs. In addition, vertical arrangement also offers innovative and novel sensory experiences. There are also various layout methods for arranging characters, including stacking characters vertically, intertwining horizontal and vertical arrangements, and sandwiching characters between lines. In general, when the text is long and contains a lot of information, horizontal arrangement of Chinese characters is more common. However, in situations that call for a fresh, minimalist, or personalized style, vertical arrangement can be an alternative, making the conveyed information more interesting and lively.

### In the end

In this study, we conducted eye-tracking experiments on glance effect of Chinese characters from the perspective of vertical and horizontal arrangements of Chinese characters. Due to the unique shape of Chinese characters, the vertical and horizontal layouts of Chinese characters in graphic-based visual communication designs have resulted in certain differences in understanding and generation of poster meanings. In history, ancient Chinese books were mainly printed in vertical layouts(and still be used by Japanese and Taiwan district’s poster and book designs presently), Currently, when it comes to the horizontal and vertical arrangement of Chinese characters in graphic design, designers generally rely on their intuitive experience to design Chinese characters in relation to graphics, considering the special structure and stroke characteristics of Chinese characters with more complex structures^46^. This study uses a combination of more objective eye-tracking experiment data and questionnaire data to conclude that, under the premise of meeting certain aesthetic requirements, the vertical layout of Chinese characters in design is more conducive to information dissemination. However, the impact on the communication of information is not limited to vertical and horizontal arrangements of Chinese characters, typeface design, stroke weight and contrast polarity may also affect the dissemination of Chinese characters on mobile phones, wearable devices, subway signs, advertisements, etc.^12^. At the same time, the spatial complexities(such as stroke numbers) of Chinese characters also leads to different applications of Chinese characters in graphic design compared to that of alphabetic languages^47^. The different combinations of graphics and text will have different impacts on information dissemination.

As the data for this experiment was collected during the pandemic, the sample size is relatively restricted. Prospective research endeavors should broaden the scope of study to include a more expansive sample and scrutinize the influences of demographic attributes(such as the elderly and the children), as well as the text layout in more experimental materials, the number of columns, font type, font size and color, on the effectiveness and efficiency of information dissemination.

## Data Availability

The data and materials supporting the findings of this study are available from the corresponding author upon reasonable request.
